# Ultrasonic vocalizations in mouse models for speech and socio-cognitive disorders: insights into the evolution of vocal communication

**DOI:** 10.1111/j.1601-183X.2010.00610.x

**Published:** 2011-02

**Authors:** J Fischer, K Hammerschmidt

**Affiliations:** † Cognitive Ethology Laboratory, German Primate CenterGöttingen, Germany; ‡ Courant Research Center “Evolution of Social Behaviour”, University of GöttingenGöttingen, Germany

**Keywords:** Autism, communication, evolution, *FOXP2*, mice, neuroligin, speech, ultrasound, vocalization

## Abstract

Comparative analyses used to reconstruct the evolution of traits associated with the human language faculty, including its socio-cognitive underpinnings, highlight the importance of evolutionary constraints limiting vocal learning in non-human primates. After a brief overview of this field of research and the neural basis of primate vocalizations, we review studies that have addressed the genetic basis of usage and structure of ultrasonic communication in mice, with a focus on the gene *FOXP2* involved in specific language impairments and neuroligin genes (*NL-3* and *NL-4*) involved in autism spectrum disorders. Knockout of FoxP2 leads to reduced vocal behavior and eventually premature death. Introducing the human variant of FoxP2 protein into mice, in contrast, results in shifts in frequency and modulation of pup ultrasonic vocalizations. Knockout of NL-3 and NL-4 in mice diminishes social behavior and vocalizations. Although such studies may provide insights into the molecular and neural basis of social and communicative behavior, the structure of mouse vocalizations is largely innate, limiting the suitability of the mouse model to study human speech, a learned mode of production. Although knockout or replacement of single genes has perceptible effects on behavior, these genes are part of larger networks whose functions remain poorly understood. In humans, for instance, deficiencies in NL-4 can lead to a broad spectrum of disorders, suggesting that further factors (experiential and/or genetic) contribute to the variation in clinical symptoms. The precise nature as well as the interaction of these factors is yet to be determined.

A classic theme in natural philosophy is the question of what distinguishes our own species from others (Wild 2008), particularly with regard to Darwin's notion of continuity in the origin of species (Darwin 1871). Although initial accounts of differentiation favored more materialistic features such as tool use or cooperative hunting (Lee & DeVore 1968), one central motif today is the ability to speak. The question of the biological origin of language quickly followed (Fitch 2010), piquing a lively debate regarding which aspects of language faculty are restricted to our own species (Fischer, in press; Hauser *et al.* 2002). Current evidence indicates that although our closest living relatives, non-human primates, largely lack volitional control over the structure of their vocalizations (Jürgens 2009), they have some control over call usage (Seyfarth & Cheney 1997). In terms of their perceptual abilities, the differences appear less pronounced (Fischer 1998; Hammerschmidt & Fischer 2008).

The purpose of the present review is to explore ways in which genetic studies in mouse models can contribute to a better understanding of the evolution of human communication. One specific aim is to elucidate the limitations in vocal communication of non-human primates. We therefore begin with a review of the vocal communication of non-human primates, including the neural circuits underlying call usage and structure. This background knowledge is essential to understand the derived features of neural circuitry in the human lineage that are seen as a precondition for vocal learning in our own species and to place the studies of mouse ultrasonic vocalizations (USVs) into an appropriate context. We begin this central part with a brief introduction to the structural and functional properties of mouse USVs, and then summarize the results of two exemplary sets of studies. The first study set focused on the effects of *FoxP2* with particular regard to its impact on structural properties of vocalizations, whereas the second study set assessed the importance of neuroligin genes on the usage of vocalizations. For comparative purposes, we will make some reference to research on bird song, another important study system to elucidate the foundations of vocal learning.

## Non-human primate vocalizations and the evolution of speech

Language in general is characterized by a set of features that distinguish it from other means of communication (Fischer, in press; Hauser *et al.* 2002; Hockett *et al.* 1960). One fundamental aspect is its symbolic nature and another the existence of a set of rules (syntax) that gives rise to novel meanings by systematic composition of the units that make up the language (Hurford 2007). Both symbolism and syntax are based on conventionalization, and hence learning plays a major role (Tomasello 2003). Spoken language is also characterized by its linear sequence (in contrast to sign languages, for instance, which operate in space and time) as well as by its use of the vocal-auditory channel (Hockett *et al.* 1960).

Comparative analyses of the communicative abilities of our closest living relatives, monkeys and apes, have constituted a productive way of approaching the language origin. One question has been whether learning is as important to the development of the species-specific communication repertoire in non-human primates as it is in humans (Egnor & Hauser 2004). Other studies have investigated whether monkey vocalizations refer to objects and events in the external world (Fischer *et al.* 1995; Seyfarth *et al.* 1980; Zuberbühler *et al.* 1999) or whether animal vocalizations include syntactic rules (Arnold & Zuberbühler 2008). Studies on the ontogeny of vocal production as well as the neurobiological foundations of vocal control in non-human primates suggest that the structure of primate vocalizations is largely innate (reviewed in Hammerschmidt & Fischer 2008). Exposure to species-specific calls and auditory feedback do not appear to be prerequisites for the proper development of the vocal repertoire. Although some developmental modifications occur, most can be attributed to growth (Ey *et al.* 2007), changes in hormone levels (Pfefferle *et al.* 2008) or arousal (Fichtel *et al.* 2001). Although calls are frequently uttered in bouts, it has been questioned whether non-human primate sequences can be described in terms of syntactic rules (Számadó*et al.* 2009).Yet, non-human primate listeners, as well as members of other taxa, appear to be apt interpreters who are able to reorganize continuous acoustic variation into discrete categories (Fischer 1998), attribute meaning to sounds (Kaminski *et al.* 2004; Seyfarth & Cheney 2003) as well as to gestures and postures (Pika *et al.* 2005), and integrate contextual and signal information when choosing an appropriate response (Rendall *et al.* 1999). In summary, the differences in the communicative abilities of non-human primates and humans are largely seen in the realm of signal production (utterance), while they are more similar in terms of comprehension where learning appears to play a role in both humans and non-human primates (Fischer 2004; Fischer *et al.* 2000; Seyfarth & Cheney 1997).

## The neural basis of sound production in non-human primates

The vocal pathway in terrestrial mammals (and other taxa) involves a number of different subsystems, contributing to different degrees in the initiation of vocalization and the structural properties of the calls. In a recent review, Jürgens (2009) proposed two separate pathways involved in the control of vocalizations. The first runs from the anterior cingulate cortex via the midbrain periaqueductal gray (PAG) into the reticular formation of pons and medulla oblongata and from there to the phonatory motoneurons. The anterior cingulate cortex is involved in the volitional control of call onset in non-human primates (Sutton *et al.* 1974) as well as in humans (Jürgens & von Cramon 1982). The midbrain PAG serves as a collector or relay station for the descending vocalization-controlling pathways, integrating incoming information and triggering specific innate vocal patterns. The PAG has therefore been ascribed as a gating function (Jürgens 2009). Electrical stimulation of this area elicits vocalizations in several species and PAG lesioning in a number of species – including squirrel monkeys, macaques, cats, rats and humans – causes muteness (reviewed in Jürgens 1994).

The second vocalization control pathway described in the Jürgens (2009) review runs from the motor cortex via the reticular formation to the phonatory motoneurons. This pathway has been shown to include two feedback loops, one involving the basal ganglia and the other involving the cerebellum (Jürgens 2009). A comparison of vocalization pathways among terrestrial mammal species has revealed that only humans exhibit a direct pathway from the motor cortex to the motoneurons controlling the larynx muscles. In contrast, connections between the limbic cortex and the motoneurons constitute an ancestral trait found in many non-human species (for reviews see Jürgens 2002, 2009). These studies also show that both pathways are linked to the different motoneurons that innervate the respective muscles for vocal fold, lip, jaw and tongue movements via the reticular formation.

The role of the basal ganglia in controlling motor output has long been recognized (Gazzaniga 2004). Recent attention has been paid to their involvement in speech production (Lieberman 2002, 2006; Ullman 2001), in particular the dopaminergic pathways involving the basal ganglia. Cortico-basal ganglia circuits in the striatum receive input from the cortex as well as dopaminergic neurons and send integrated signals to brain stem structures as well as feedback loops back to the cortex (Graybiel 2008). Reduced dopamine release in the striatum is positively correlated with speed and accuracy of phonological processing (Tettamanti *et al.* 2005), parts of the striatum are involved in lexical-semantic control (Crinion *et al.* 2006), and, depending on the subregions involved, patients with Huntington's disease have difficulties in the recovery of lexical information and the application of combinatorial rules (Teichmann *et al.* 2008). Cortico-basal ganglia circuits, including their dopaminergic modulations, are also crucial for song learning in birds (Hara *et al.* 2007; Jarvis 2004). Jarvis (2004) suggested a possibly more important parallel vis-à-vis the neural basis of sound production, pointing out that the songbird and parrot posterior vocal pathways are similar in connectivity to mammalian motor corticospinal pathways.

The most important derived feature in the human lineage regarding the ontogeny of speech appears to be the evolution of the direct pathway from the motor cortex to the motoneurons, enabling volitional control over the oscillations of the vocal folds. Together with the intricate coordination of breathing and articulation, this feature allows for the precise control over speech production. The role of the basal ganglia in the modulation of vocal behavior, in contrast, appears to be an ancestral feature. The detailed investigations of the brain mechanisms underlying vocal control now call for the elucidation of the genes that might be involved in the reorganization of the brain that enabled humans to talk (Fisher & Marcus 2006).

## Structure and function of mouse USVs

USVs occur in a wide range of taxa such as rats (Brudzynski 2005; Kaltwasser 1990; Knutson *et al.* 2002; Schwarting *et al.* 2007) and other rodents (Sales 1972; Sewell 1967; Wilson & Hare 2004) as well as bats (Russ *et al.* 2004) and frogs (Arch *et al.* 2009). In the following, we will focus on USVs in mice.

Interest in mouse vocal behavior goes back quite some time (reviewed in Nyby 2001; Sewell 1967, 1970; Whitney *et al.* 1973). One of the most widely studied vocalizations in mice is the isolation call of pups. These calls can be elicited reliably when young pups are either isolated from their mother or during temperature stress (Ehret 2005; Hahn & Schanz 2005; Hahn *et al.* 1998; Sales & Smith 1978). These studies have also verified that these USVs are not simply by-products of motor activity or physiological maneuvering such as abdominal compression (Blumberg 2000a, b) and can be seen as biologically meaningful signals (Ehret 2005). In addition, several playback studies have shown that isolation calls alone are able to elicit searching behavior by mothers (for instance, Ehret & Haack 1982; Hahn & Lavooy 2005; Uematsu *et al.* 2007).

Calling rate and structure of USVs are largely dependent on age. Calling rate shows a U-shaped function with a peak of calls between 7 and 9 days (Hahn & Schanz 2005; Hahn *et al.* 1998). Call duration declines with age, whereas call pitch increases (Hahn *et al.* 1998; Sales & Smith 1978). Genetic differences between various strains also seem to influence call rate, duration and frequency characteristics of isolation calls (Hahn & Schanz 2005; Nietschke *et al.* 1972). In recent years, the occurrence and structure of pup isolation calls have come to be recognized as an informative readout in translational studies as well as in studies of the genetic basis of social and communicative behavior.

Although the investigation of mouse pup isolation calls has been ongoing for several decades, a recent study by Holy & Guo (2005) has sparked the attention of the broader research community and the public alike. Holy and Guo advanced the view that these vocalizations function as courtship displays. By incorporating the temporal and spectral features of male mouse vocalizations, Holy and Guo were able to sort the structurally highly variable call elements into a few discrete categories using as a criterion the temporal location of a major frequency jump within a call element. They were also able to show that the succession of call elements or syllables differs significantly from a random pattern, with preferred transition probabilities between different syllable types. Based on these findings, they suggested that male mouse courtship vocalizations are structurally, ontogenetically and functionally comparable to bird song. To further explore this conjecture, a brief excursion into the literature on bird song appears to be in order.

Bird song may be produced by both males and females or one sex only. In the majority of cases, males produce the song, but there are intriguing exceptions of sex role reversal where the females do the singing (Geberzahn *et al.* 2009; Langmore 1998). Although duetting occurs more frequently in tropical regions, male solo singing is the typical pattern in temperate regions (Catchpole & Slater 2008). In terms of structure, songs are typically more complex than calls, with several notable exceptions. Grasshopper warblers, for instance, produce song that consists of a continuous repetition of one single syllable (Catley 1986). As a result of the high variety of complexity in bird songs, it is difficult to use structural features as a basis for comparison. To term sequences of mouse vocalizations as ‘song’ is appropriate if one defines song as lengthy bouts of calling. This definition can also encompass courtship songs of anurans and insects (Gerhardt 1981; Gerhardt & Huber 2002) and those of baleen whales (Payne & McVay 1971). In terms of ontogenetic development, bird song appears to be learned. For birds in temperate regions, nestlings hear the song of their father or other males in the vicinity and form a so-called ‘template’. During the fall in the wintering grounds, the young birds begin to vocalize softly, called ‘sub-song’. Adult song structures emerge gradually during practice until the following spring when these male birds are able to produce fully crystallized versions of their song (Hultsch & Todt 1989). Species differ in terms of their predisposition to attend to their own specie's song, but in all cases auditory input is crucial for the formation of full song (reviewed in Catchpole & Slater 2008). In contrast, the USVs of mice are considered to be innate. Whether auditory feedback plays a role in the formation of the species-specific vocalization is a matter of debate, but it seems quite likely that in mice – as in most other terrestrial mammals – auditory input is not a prerequisite for the development of vocal patterns. In terms of functionality, there is ample evidence that male (and female) birds sing to attract a mating partner, whereas both solo song and duetting serve to establish and defend territories (Catchpole & Slater 2008).

**Figure 1:**
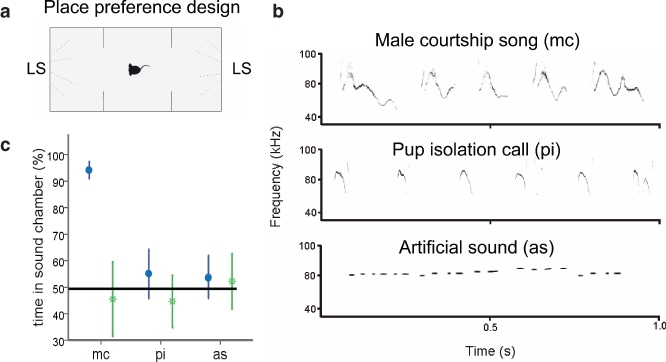
Playback experiments with mice (a) Sketch of place preference design. LS = loudspeaker. In the two trials, females were presented with songs from different males, all of which were unfamiliar. (b) Spectrograms of playback sounds. (c) Percentage of time the females spent in the chamber with the playback sound (mean ± SEM). Line indicates chance level. Filled squares = first presentation, open squares = second presentation (N = 32) (figure adapted from Hammerschmidt *et al.* 2009).

To elucidate the function of male mouse USVs, we conducted playback experiments to assess female mice responses to male mouse ‘song’ (Hammerschmidt *et al.* 2009). We used a place preference design to test whether male song alone can evoke approach behavior (Fig. 1a). For control sounds, we presented ultrasonic pup vocalizations and artificial sounds (Fig. 1b). Because some studies have suggested that the female reproductive state can influence their response behavior (Byatt & Nyby 1986), we tested each female during estrus and diestrus. We predicted that both control stimuli (pup vocalization and artificial sound) would not evoke approach behavior, based on a previous study that showed that only lactating females respond to playback of pup sounds (Uematsu *et al.* 2007).

Female mice are attracted to playback sounds of male sounds (Fig. 1c), in-line with the results of the study by Pomerantz and colleagues (Pomerantz *et al.* 1983) which showed that females prefer intact vocalizing males over devocalized males. Wild female mice are similarly attracted to male USVs (Musolf *et al.* 2009), and their responses to male song are similar to those of female birds and other species. Field playback experiments as well as laboratory studies have shown that the song alone is sufficient to elicit approach behavior of females to the sound source (Baker *et al.* 1981; Eriksson & Wallin 1986). In contrast, females in our study did not respond to pup vocalizations or to artificial sounds (Figs. 1b,c), indicating that females can differentiate between male song and other USV in this frequency range. Contrary to our predictions, the reproductive state of females had no influence in their response to the playback sounds. In addition, females habituated rapidly to the presentation of the control stimuli. Females responded only the first time to the playback of male songs (Fig. 1c). A second playback 2 or 3 days later evoked no responses (see Shepard & Liu, in press for similar observations). This is in stark contrast to findings in other taxa where courtship vocalizations evoke sustained responses (Catchpole *et al.* 1984; Gerhardt 1991, 1994; Scheuber *et al.* 2004; Searcy & Marler 1981). Because the females in our mouse model responded only the first time to the male song, it is unlikely that male mice vocalizations function as courtship song to attract females over larger distances – a scenario that would require several song-and-response iterations. It seems more likely that male mouse song is used to facilitate close body contact with females for mating purposes.

Although the focus in recent years has been on male mouse USV, earlier reports showed that female mice use similar vocalizations. Sales (1972) was the first to describe 70 kHz ultrasound vocalizations when he put females together in one cage, a finding later confirmed by Maggio & Whitney (1985). These authors hypothesized that the female vocalizations served to establish dominance hierarchies within demes. However, at the same time they found that the presence of males inhibited USV of females, a puzzling result given that wild mouse demes include males. In addition, studies that have tried to elicit USV using chemosensory cues in female mice have been not as successful as similar studies in males (Maggio & Whitney 1985; Nyby 2001). Possibly, these negative results contributed to the fact that female USV did not attract much further attention.

Two recent studies that used the resident-intruder paradigm to elicit USV from female mice yielded more promising results. In one design, resident animals were separated for one or more days in a ‘home’ cage. Following the separation, another animal – the intruder – was placed in the home cage of the resident animal. Moles and colleagues (2007) were able to show that females emit USVs during social encounters with intruding females. The number of calls seemed to be modulated by the motivational state of the emitter during the estrous cycle, and there was a positive correlation between the number of calls and the time spent by the resident sniffing the intruder female (Moles *et al.* 2007). In general, these results confirmed that USVs emitted during such social interaction can be used as an indicator of social recognition, and therefore as a dependent variable to detect disruption of social memory in mice. Scattoni *et al.* (2008a, 2009) confirmed these findings by showing that the USV produced during resident-intruder test could be used to characterize social relationship between different females.

The above overview describing some key questions in the evolution of language debate, as well as the most significant features of mouse USVs, serves as the framework for the following section that reviews exemplary studies addressing specific genes that have been implicated in language impairments and socio-cognitive deficits.

## The importance of *FOXP2* for speech and language

The *FOXP2* gene was identified in a British family whose specific language impairments appeared to be inherited in an autosomal dominant fashion (Hurst *et al.* 1990). Initially, linkage was found to a region of chromosome 7. Subsequently, a case of chromosome translocation was found in an unrelated patient with a similar phenotype, allowing geneticists to eventually identify a point mutation in the *FOXP2* gene (Fisher & Marcus 2006). Strikingly, *FoxP2* appears to be highly conserved [for an exception to this discovered in bats by Li and colleagues (2007); see below]. Analyses of the evolution of the *FoxP2* gene in primates have identified two amino acid substitutions (T303N, N325S) believed to have become fixed in the human lineage after its separation from the chimpanzee and which appear to have been subject to positive selection (Enard *et al.* 2002; Zhang *et al.* 2002). These findings gave rise to the notion that these substitutions underwent selection due to effects on some aspects of speech and language (Fisher & Scharff 2009).

In humans, when one allele carries a missense mutation (R553H) affecting the DNA binding domain of the protein, is truncated due to a nonsense mutation (R328X) or is disrupted by a chromosomal rearrangement, the development of speech and language is impaired (Lai *et al.* 2001). Importantly though, *FOXP2* is not a language gene but a transcription factor that affects the function of many genes and is involved, for instance, in the development of the lungs, heart and other organs (Fisher & Marcus 2006). Its precise effects in the phenotype affecting language development have been a matter of some debate. Affected individuals have problems with sequential speech production that can lead to major problems with intelligibility. They also have more general difficulties with language, made evident in their written language and in language comprehension (Bishop 2009).

To study the effects of variants in the corresponding FoxP2 protein on vocal behavior, two taxa have been studied in greater detail, namely mice and song birds. Mice have been established as model mammalian organisms in numerous genetic studies (Fisher & Scharff 2009), whereas songbirds are of specific interest because song is learned and thus constitutes a valuable analogy for speech which is also a learned mode of production. The anatomical validity of this analogy is reinforced by findings that songbirds and humans express FoxP2 in comparable and homologous brain areas, including the striatum and primary sensory nuclei of the thalamus (Haesler *et al.* 2004; Teramitsu *et al.* 2004). In songbirds, lentivirus-mediated RNA interference (RNAi) to reduce FoxP2 levels in brain region Area X results in imprecise song copying (Haesler *et al.* 2007). This brain region has been identified as part of the songbird basal ganglia dedicated to song (Doupe *et al.* 2005; Jarvis *et al.* 2005), containing medium spiny neurons similar to mammalian basal ganglia (Farries *et al.* 2005). *FoxP2* was shown to be differentially upregulated in this area in zebra finches when the birds were learning to sing their song (Haesler *et al.* 2004). In adult birds, *FoxP2* was acutely downregulated in Area X, but only in males who sang by themselves (undirected singing) and not in males who sang to females (directed singing) (Teramitsu & White 2006); this was true in both hearing and deaf birds (Teramitsu *et al.* 2010).

Other comparative data on *FoxP2* support the view that this gene is closely linked to vocal behavior. The different clades of echolocating bats show significant changes in the *FoxP2* gene sequence (Li *et al.* 2007). This is surprising as *FoxP2* has a remarkably conserved profile in other mammals. Li and colleagues (2007) hypothesized that this pattern of gene modification is related to the fact that bats rely on extremely precise vocalizations for predation. Mice homozygous for non-functional *FoxP2* alleles produce significantly fewer isolation calls than their wild-type (WT) littermates (Fujita *et al.* 2008; Groszer *et al.* 2008; Shu *et al.* 2005) – although, importantly, these studies did not report any structural differences in the properties of the calls. However, these mice exhibit severe developmental deficits and die around 3 weeks after birth, implying that the reduction in ultrasonic vocalization might not represent specific effects of FoxP2 on mouse vocalizations (Groszer *et al.* 2008). Mouse pups with heterozygous non-functional *FoxP2* alleles reportedly have mild developmental delays and produce fewer ultrasonic calls (Fujita *et al.* 2008; Shu *et al.* 2005).

**Figure 2:**
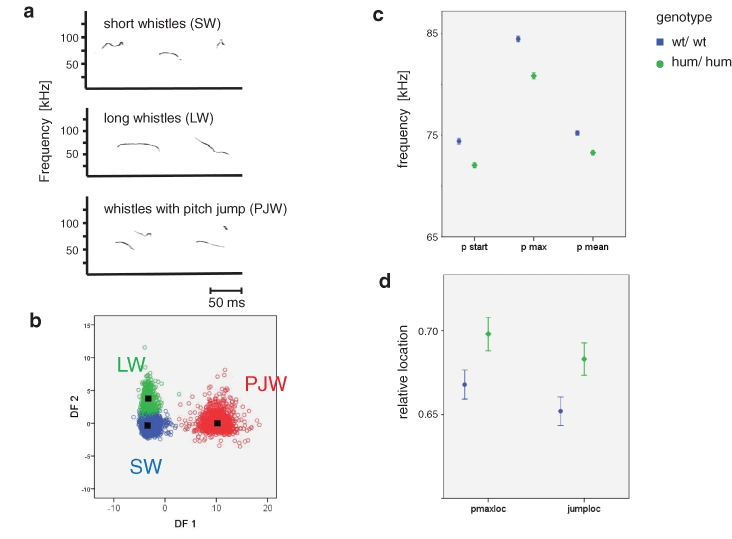
Characterization of pup isolation calls (a) Spectrograms of the three major call types. (b) Scattergram derived from a discriminant function analysis that depicts the categorization of call types. Discriminant function 1 (DF1) is mainly correlated with maximum pitch jump, DF2 with call duration. The discriminant function was calculated based on data published in Enard *et al.* (2009). (c) Acoustic differences in relation to genotype for short whistles (SW) and long whistles (LW). The p start, p max and p mean = start, maximum and mean of the peak frequency. In whistle-like calls, the peak frequency corresponds to fundamental frequency whereas the harmonics have such low amplitude that they are often not visible. (d) Acoustic differences related to genotype for whistles with pitch jump (PJW). P maxloc = location of maximum peak frequency in relation to call duration, calculated as coefficient ranging between 0 and 1. Jumploc = location of the highest change in peak frequency, also measured in relation to call duration (N: *FoxP*2^*WT/WT*^ = 39; *FoxP*2^*hum/hum*^ = 32). (c) and (d) are redrawn from Enard *et al.* (2009).

To study the effects of the human version of the *FoxP2* gene in a mouse model, a large consortium of researchers led by Wolfgang Enard and Svante Pääbo from the Max-Planck-Institute for evolutionary Anthropology in Leipzig genetically engineered a mouse in which the two amino acid replacements had been introduced to mimic the human variant of the *FoxP2* gene (Enard *et al.* 2009). Mice carrying the *FoxP2*^*hum*^ allele were generated from C57BL/6 ES cell clones. As the FoxP2 protein in chimpanzees differs from FoxP2 in mice by only one conservative amino acid substitution (D80E); the WT mouse FoxP2 protein can be used as a model for the ancestral version of the human FoxP2 protein (Enard *et al.* 2009). Pup isolation calls using this engineered strain were recorded on postnatal day P4, P7, P10 and P13. All genotypes showed the normal age-dependent changes. Older mice (P10, P13) produced fewer isolation calls with structural differences of increased duration and pitch. There were no differences between genotypes with regard to the number of calls or the call duration or in terms of the temporal structure of call sequences, and both genotypes were able to produce all vocal types. Figure 2a shows examples of the three main vocal types. Mice isolation calls have a highly variable structure with numerous intermediate calls difficult to assign to a specific category. The only unambiguous category was the one containing whistles with pitch jumps higher than approximately 15 kHz (Fig. 2b). All other parameters showed a continuous distribution. Despite this general similarity of vocal repertoire and usage of calls, our acoustic analysis showed significant differences in call structure. In *FoxP2*^*hum*^ mice, the calls had a lower start peak frequency with lower mean, minimum and maximum peak frequencies (Fig. 2c). In addition, there were significant differences in the location of the maximum of the peak frequency and in the location where pitch jumps occur (Fig. 2d). *FoxP2*^*hum*^ mice also showed reduced dopamine concentrations in the brain, indicating that the humanized *FoxP2* allele affects the basal ganglia. This was further evidenced by the medium spiny neurons in the striatum that had longer dendrites and showed an increased synaptic plasticity (Enard *et al.* 2009). In contrast to the previously mentioned studies using non-functional *FoxP2* alleles, *FoxP2*^*hum*^ influenced ultrasonic vocalization of pups in a specific fashion (Enard *et al.* 2009). This influence was subtle and within the range of normal variation among mice, raising the question of which specific aspect of sound production was actually affected.

Sound production in terrestrial mammals is generally based on the production of an air stream with the lungs that induces vocal fold oscillations in the larynx (Fitch 2000; Hammerschmidt & Fischer 2008; Lieberman & Blumstein 1988). Although ultrasonic vocalizations in rodents are also produced in the larynx, they are thought to derive from an aerodynamic whistle rather than vibrations of vocal cords (Roberts 1975). This means that further studies will be needed to identify homologies and analogies in the neural circuitry underlying USVs in mice and vocalizations in non-human primates and humans in order to more critically assess to what extend mouse vocalizations can model aspects of human speech and language evolution. In conjunction with work on other taxa such as song birds, it might eventually be possible to unravel the role that FoxP2 plays in vocal production in human and non-human species alike. For example, work to detect *FoxP2* gene targets and networks in songbirds can be compared with targets and networks in humans and thus highlight shared and unique subsets (Fisher & Scharff 2009). Shared gene networks are hypothesized to be involved in vocal mimicry and sequential learning in the vocal motor domain. A further complication when relating this research to human vocal production is the occurrence of differential expression rates in different target cells in humans (Konopka *et al.* 2009). In spite of all these limitations, we believe that the study of FoxP2 provides one of the most promising research avenues for gaining a better understanding of the role of particular genes on complex social behaviors and in the evolution of speech.

## Variation in call usage and regulation of social behavior

A number of genes have been implicated in the variation of USV call usage in mice, specifically genes that are involved in the regulation of social behavior. Two classic factors that modulate social interaction, social recognition, pair bonding and parental care are oxytocin (OXT) and vasopressin – hypothalamic neuropeptides excreted by the neurohypophysis (Lim & Young 2006; Neumann 2008). Mice lacking the OXT gene show impaired social memory (Ferguson *et al.* 2000) and deficits in maternal behavior (Pedersen *et al.* 2006). Oxytocin knockout (KO) infant mice were less vocal than the corresponding WT controls, and male mice were generally more aggressive and less fearful in a plus-maze test (Winslow *et al.* 2000). Vasopressin also appears to be involved in the motivational aspects of vocal communication: vasopressin-1b KO mice, for instance, were found to produce fewer ultrasonic vocalizations (Scattoni *et al.* 2008b).

The usage of ultrasonic vocalizations appears to also be influenced by the dopaminergic reward system involved in a variety of behaviors, including affective responses, positive reinforcement, foraging and sexual behavior (Schultz 2006). To give just one example, a recent study by Wang and colleagues (2008) investigated the effects of knockout of D2 receptors of the dopaminergic system as well as of knockout of three types of muscarinic receptors on the usage and structure of male mouse USVs in the mating context. They found no effect for knockout of D2 on call usage, but a slight effect on call duration. Interestingly though, knockout of muscarinic receptors of the cholinergic system, which plays an important role in modulating functions of the dopaminergic systems in the brain, did have an effect on both the structure and the usage of vocalizations: M2 and M5 KO mice produced fewer, and disproportionally fewer frequency modulated calls, whereas knockout of M4 had no effect. In addition, knockout of M2 and M5 led to the production of calls with a lower peak frequency. The authors suggested that muscarinic receptors influence male USV production via dopamine activation (Wang *et al.* 2008).

As mentioned early on, our research interest lies in the elucidation of the evolution of communicative behavior with special emphasis on the evolution of speech. Because of the link between communicative behavior and the development of perspective taking and mental state attribution in human children, genes that have been implicated in autism spectrum disorders (ASD) are of particular interest. Typical symptoms of ASD are social deficits such as impairments in the ability to take the perspective of others, language deficits, as well as restricted interests (Baron-Cohen *et al.* 2000). A number of monogenic heritable autism spectrum condition forms have been shown to be caused by loss-of-function mutations in genes that code for synaptic cell adhesion proteins such as the neuroligin and neurexin genes and genes that encode synaptic scaffold proteins such as SHANK3 (The Autism Genome Project Consortium 2007). These findings indicate that aberrant signaling is involved in the etiology of ASD. More specifically, loss-of-function mutations in the genes encoding neuroligin-4 (NL-4) and point mutations in neuroligin-3 (NL-3) identified as sources of monogenic heritable ASD (Jamain *et al.* 2003). Very recent studies have shown that a complete elimination of NL-3 or NL-4 expression in mice leads to a cluster of symptoms, which are reminiscent of ASD (Jamain *et al.* 2008, Radyushkin *et al.* 2009). In particular, NL4-KO mice showed no differences in terms of memory, learning, hearing, locomotor activity, as well as a number of other behavioral assays. However, unlike their WT littermates, KO mice did not respond differentially to an intruder mouse compared with an empty compartment, suggesting a lack of salience of the social stimulus (Jamain *et al.* 2008). The volume of the cerebellum and the brain stem in NL4-KO mice was significantly smaller compared with WT controls. Overall, NL3-KO mice showed a similar pattern of deficiencies. In addition, they exhibited olfactory deficits. Remarkably, a similar phenotype is also present in a subgroup of human ASD patients (Radyushkin *et al.* 2009).

**Figure 3:**
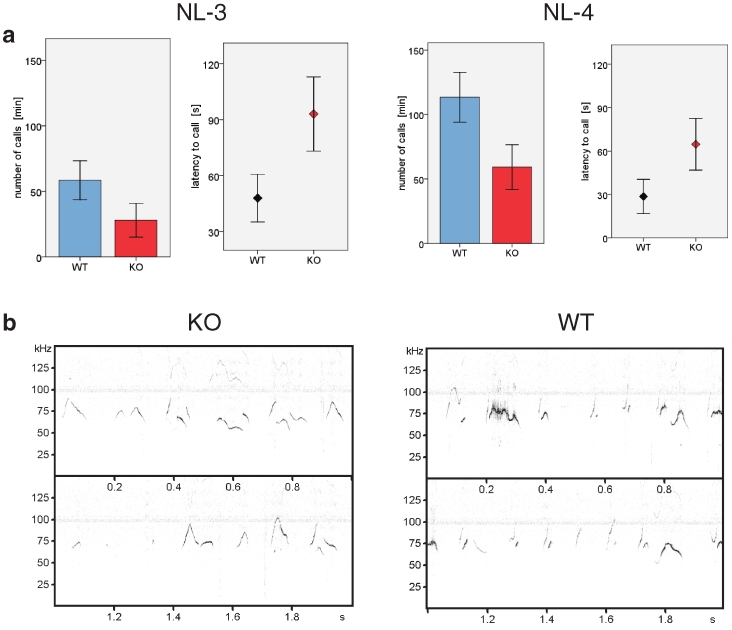
Differences in the ultrasound vocalization of NL-3 and NL-4 KO mice (a) Differences in number of calls and latency to call of male mice courtship vocalizations (N: NL-3WT = 25; KO = 5; NL-4WT = 20; KO = 16). Figure compiled from data presented in (Jamain *et al.* 2008; Radyushkin *et al.* 2009). (b) Frequency spectrogram demonstrating the possibility to produce similar call types, WT = wild-type male, KO = NL-4KO male.

We focused on the vocal communication of NL-3 and NL-4 KO mice and analyzed the ultrasonic vocalizations of male mice during courtship behavior. We found in both cases a significant reduction in the number of USV calls (Fig. 3a). Indeed, many of the knockout males did not vocalize at all. In addition, knockout males showed a significantly longer latency until they started to vocalize. Note that the relative high variation of the latencies is probably due to the fact that a few of the corresponding WT mice were not motivated to engage in courtship behavior with the females. The number of calls emitted by the mice in the two study models (NL-4 vs. NL-3) differed considerably (Fig. 3a), with NL-4 KO mice calling at about the same rate as NL-3 WT mice. The NL-4 mice were bred in the C57BL/6 strain, whereas the mice used in the NL-3 study model were bred in the C57BL/6NCrl strain. Whether this variation is due to strain differences, differences in rearing, differences in experimental conditions or in some other factor needs further investigation.

The rare cases where the KO male mice uttered calls showed that both WT and KO mice were able to produce the same call types (Fig. 3b). Although most of the KO mice did not produce calls with a long duration and/or a complex frequency structure, these calls were found to be sporadically produced by KO mice. These results indicate that NL-3 and NL-4 KO mice are in principle able to generate these calls, but that they are rather less consistent in their behavioral responsiveness to the social stimuli eliciting the calling. In summary, both study models constitute valuable analogies to some specific forms of ASD.

Other mouse models for autism have reported a very different pattern in terms of the structural property of calls. Mice of the BTBR T+ tf/J strain, which exhibit social abnormalities and repetitive behaviors, were found to have an abnormal vocal repertoire (Scattoni *et al.* 2008a). In addition, they produced a higher number and longer duration of calls than the control group. Even so, the results of the studies of mice USVs reported here support the view expressed by the Scattoni *et al.* (2008a) study that communicative behavior in mice, particularly their vocalizations, constitutes a useful assay for studying impairment of social behavior, including autism. Future research needs to address whether changes in the motivation to communicate are due to impairment in the recognition of others as salient stimuli or a specific reduction in the motivation to interact.

## Conclusions

The case studies reviewed here indicate that the ultrasonic vocalizations of mice appear to constitute a valuable readout in studies of the genetic foundations of social and communicative behavior, perhaps even giving some preliminary clues to the evolution of speech. Call rates, durations and response consistencies, in particular, appear to be sensitive variables in studies of genes involved in the modulation of social behavior. However, to date the interaction of different factors that contribute to variation in the propensity to vocalize remains largely unclear.

Before we can fully understand how different genes contribute to changes in the structure of vocalizations, we need to develop a better understanding of the sound production mechanisms. For instance, how are mouse calls with ‘pitch jumps' being produced, what role do non-linear phenomena play and what is the contribution of the vocal tract filtering (Fitch *et al.* 2003)? Furthermore, we suspect a relationship between the intensity of calling and the acoustic properties of single call elements; additional studies will be needed to put this conjecture on firm empirical grounds.

Despite the present optimism regarding the value of ultrasonic vocalizations in transgenic mice as readouts in clinical studies, some important restrictions apply in terms of their applicability to study the foundations of human speech. It is of utmost importance to be aware of the differences in the neural circuitry underlying innate vs. learned vocalizations. In other words, in the FoxP2 studies in mice reviewed here, the effects of genes are studied largely in the context of innate behavior. The ultimate goal is to understand a learned mode of vocalization production because only in this context will we enhance our understanding of the origins of speech. The finding that laboratory-produced mice carrying the human variant of the *FoxP2* gene show significant differences in the local architecture of the striatum is in-line with the view that this area is important in the fine-grained control of motor behavior. However, in addition to changes at the synaptic and local level, there is also a global reorganization of the fiber tracts that connect the brain areas involved in motor sound production and perception (Friederici 2009).

We are just beginning to grasp the complexity of the genetic networks contributing to regulations between vocal and social behavior. Studies on the genetic foundation of mouse ultrasonic vocalizations can help to put some pieces of the puzzle of language evolution in the proper place. At the same time, other issues such as the understanding of the link between mental state attribution and language and its role in the evolution of speech still remain largely elusive.

## References

[b1] Arch VS, Grafe TU, Gridi-Papp M, Narins PM (2009). Pure ultrasonic communication in an endemic Bornean frog.. PLoS One.

[b2] Arnold K, Zuberbühler K (2008). Meaningful call combinations in a non-human primate.. Curr Biol.

[b3] Baker MC, Spitlernabors KJ, Bradley DC (1981). Early experience determines song dialect responsiveness of female sparrows.. Science.

[b4] Baron-Cohen S, Ring HA, Bullmore ET, Wheelwright S, Ashwin C, Williams SCR (2000). The amygdala theory of autism.. Neurosci Biobehav R.

[b5] Bishop DVM, Bickerton D, Szathmary E (2009). What can developmental language impairment tell us about the genetic basis of syntax?. Biological Foundations and Origin of Syntax.

[b6] Blumberg MS, Sokoloff G, Kent KJ (2000a). Developmental analysis of clonidine's effects on cardiac rate and ultrasound production in infant rats.. Dev Psychobiol.

[b7] Blumberg MS, Sokoloff G, Kirby RF, Kent KJ (2000b). Distress vocalization in infant rats: what's all the fuzz about?. Psychol Sci.

[b8] Brudzynski SM (2005). Principles of rat communication: quantitative parameters of ultrasonic calls in rats.. Behav Genet.

[b9] Byatt S, Nyby J (1986). Hormonal-regulation of chemosignals of female mice that elicit ultrasonic vocalizations from males.. Horm Behav.

[b10] Catchpole CK, Slater PJB (2008). Bird Song. Biological Themes and Variations.

[b11] Catchpole CK, Dittami J, Leisler B (1984). Differential responses to male song repertoires in female songbirds implanted with estradiol.. Nature.

[b12] Catley GP (1986). Grasshopper warbler behavior when singing.. British Birds.

[b13] Crinion J, Turner R, Grogan A, Hanakawa T, Noppeney U, Devlin JT, Aso T, Urayama S, Fukuyama H, Stockton K, Usui K, Green DW, Price CJ (2006). Language control in the bilingual brain.. Science.

[b14] Darwin C (1871). The Descent of Man, and Selection in Relation to Sex.

[b15] Doupe AJ, Perkel DJ, Reiner A, Stern EA (2005). Birdbrains could teach basal ganglia research a new song.. Trends Neurosci.

[b16] Egnor SER, Hauser MD (2004). A paradox in the evolution of primate vocal learning.. Trends Neurosci.

[b17] Ehret G (2005). Infant rodent ultrasounds – a gate to the understanding of sound communication.. Behav Genet.

[b18] Ehret G, Haack B (1982). Ultrasound recognition in house mice: key stimulus configuration and recognition mechanisms.. J Comp Physiol A.

[b19] Enard W, Przeworski M, Fisher SE, Lai CSL, Wiebe V, Kitano T, Monaco AP, Paabo S (2002). Molecular evolution of FOXP2, a gene involved in speech and language.. Nature.

[b20] Enard W, Gehre S, Hammerschmidt K (2009). A humanized version of Foxp2 affects cortico-basal ganglia circuits in mice.. Cell.

[b21] Eriksson D, Wallin L (1986). Male bird song attracts females – a field experiment.. Behav Ecol Sociobiol.

[b22] Ey E, Pfefferle D, Fischer J (2007). Do age- and sex-related variations reliably reflect body size in non-human primate vocalizations – a review.. Primates.

[b23] Farries MA, Ding L, Perkel DJ (2005). Evidence for “direct” and “indirect” pathways through the song system basal ganglia.. J Comp Neurol.

[b24] Ferguson JN, Young LJ, Heam EF, Matzuk MM, Insel TR, Winslow JT (2000). Social amnesia in mice lacking the OXT gene.. Nat Genet.

[b25] Fichtel C, Hammerschmidt K, Jurgens U (2001). On the vocal expression of emotion. A multi-parametric analysis of different states of aversion in the squirrel monkey.. Behaviour.

[b26] Fischer J (1998). Barbary macaques categorize shrill barks into two call types.. Anim Behav.

[b27] Fischer J (2004). Emergence of individual recognition in young macaques.. Anim Behav.

[b28] Fischer J, Frey U, Störmer C, Willführ K Nothing to talk about? On the linguistic abilities of nonhuman primates (and some other animal species). Homo Novus–A Human Without Illusions.

[b29] Fischer J, Hammerschmidt K, Todt D (1995). Factors affecting acoustic variation in Barbary macaque (*Macaca sylvanus*disturbance calls.. Ethology.

[b30] Fischer J, Cheney DL, Seyfarth RM (2000). Development of infant baboons' responses to graded bark variants.. P Roy Soc Lond B Bio.

[b31] Fisher SE, Marcus GF (2006). The eloquent ape: genes, brains and the evolution of language.. Nat Rev Genet.

[b32] Fisher SE, Scharff C (2009). FOXP2 as a molecular window into speech and language.. Trends Genet.

[b33] Fitch WT (2000). The evolution of speech: a comparative review.. Trends Cogn Sci.

[b34] Fitch WT (2010). The Evolution of Language.

[b35] Fitch WT, Hauser MD, Simmons AM, Popper AN, Fay RR (2003). Unpacking “Honesty”: Vertebrate Vocal Production and the Evolution of Acoustic Signals. Animal Communication.

[b36] Friederici AD (2009). Allocating functions to fiber tracts: facing its indirectness.. Trends Cogn Sci.

[b37] Fujita E, Tanabe Y, Shiota A, Ueda M, Suwa K, Momoi MY, Momoi T (2008). Ultrasonic vocalization impairment of FoxP2 (r552h) knockin mice related to speech-language disorder and abnormality of purkinje cells.. Proc Natl Acad Sci USA.

[b38] Gazzaniga M (2004). The Cognitive Neurosciences. Bradford Books.

[b39] Geberzahn N, Goymann W, Muck C, Ten Cate C (2009). Females alter their song when challenged in a sex-role reversed bird species.. Behav Ecol Sociobiol.

[b40] Gerhardt HC (1981). Mating call recognition in the barking tree frog (*Hyla gratiosa*: responses to synthetic calls and comparisons with the green tree frog (*Hyla cinerea*.. J Comp Physiol.

[b41] Gerhardt HC (1991). Female mate choice in treefrogs: static and dynamic acoustic criteria.. Anim Behav.

[b42] Gerhardt HC (1994). The evolution of vocalization in frogs and toads.. Annu Rev Ecol Syst.

[b43] Gerhardt HC, Huber F (2002). Acoustic Communication in Insects and Anurans: Common Problems and Diverse Solutions.

[b44] Graybiel AM (2008). Habits, rituals, and the evaluative brain.. Annu Rev Neurosci.

[b45] Groszer M, Keays DA, Deacon RMJ (2008). Impaired synaptic plasticity and motor learning in mice with a point mutation implicated in human speech deficits.. Curr Biol.

[b46] Haesler S, Wada K, Nshdejan A, Morrisey EE, Lints EKT, Jarvis ED, Scharff C (2004). FoxP2 expression in avian vocal learners and non-learners.. J Neurosci.

[b47] Haesler S, Rochefort C, Georgi B, Licznerski P, Osten P, Scharff C (2007). Incomplete and inaccurate vocal imitation after knockdown of Foxp2 in songbird basal ganglia nucleus area x.. Plos Biol.

[b48] Hahn ME, Lavooy MJ (2005). A review of the methods of studies on infant ultrasound production and maternal retrieval in small rodents.. Behav Genet.

[b49] Hahn ME, Schanz N (2005). The effects of cold, rotation, and genotype on the production of ultrasonic calls in infant mice.. Behav Genet.

[b50] Hahn ME, Karkowski L, Weinreb L, Henry A, Schanz N, Hahn EM (1998). Genetic and developmental influences on infant mouse ultrasonic calling. II. Developmental patterns in the calls of mice 2–12 days of age.. Behav Genet.

[b51] Hammerschmidt K, Fischer J, Griebel U, Oller K (2008). Constraints in primate vocal production. The Evolution of Communicative Creativity: From Fixed Signals to Contextual Flexibility.

[b52] Hammerschmidt K, Radyushkin K, Ehrenreich H, Fischer J (2009). Female mice respond to male ultrasonic ‘songs’ with approach behaviour.. Biol Lett.

[b53] Hara E, Kubikova L, Hessler NA, Jarvis ED (2007). Role of the midbrain dopaminergic system in modulation of vocal brain activation by social context.. Eur J Neurosci.

[b54] Hauser MD, Chomsky N, Fitch WT (2002). The faculty of language: what is it, who has it, and how did it evolve?. Science.

[b55] Hockett CF, Lanyon WE, Tavolga WN (1960). Logical considerations in the study of animal communication. Animal Sounds and Communication.

[b56] Holy TE, Guo Z (2005). Ultrasonic songs of male mice.. Plos Biol.

[b57] Hultsch H, Todt D (1989). Song acquisition and acquisition constraints in the nightingale, *Luscinia megarhynchos*. Naturwissenschaften.

[b58] Hurford JR (2007). On the Origins of Meaning–Language in the Light of Evolution.

[b59] Hurst JA, Baraitser M, Auger E, Graham F, Norell S (1990). An extended family with a dominantly inherited speech disorder.. Dev Med Child Neurol.

[b60] Jamain S, Quach H, Betancur C, Rastam M, Colineaux C, Gillberg IC, Soderstrom H, Giros B, Leboyer M, Gillberg C, Bourgeron T, Paris Autism Res Int Sibpair Study. (2003). Mutations of the X-linked genes encoding neuroligins NLGN3 and NLGN4 are associated with autism.. Nat Genet.

[b61] Jamain S, Radyushkin K, Hammerschmidt K, Granon S, Boretius S, Varoqueaux F, Ramanantsoa N, Gallego J, Ronnenberg A, Winter D, Frahm J, Fischer J, Bourgeron T, Ehrenreich H, Brose N (2008). Reduced social interaction and ultrasonic communication in a mouse model of monogenic heritable autism.. Proc Natl Acad Sci USA.

[b62] Jarvis ED (2004). Learned birdsong and the neurobiology of human language.. Ann N Y Acad Sci.

[b63] Jarvis ED, Gunturkun O, Bruce L (2005). Avian brains and a new understanding of vertebrate brain evolution.. Nat Rev Neurosci.

[b64] Jürgens U (1994). The role of the periaqueductal grey in vocal behaviour.. Behav Brain Res.

[b65] Jürgens U (2002). Neural pathways underlying vocal control.. Neurosci Biobehav R.

[b66] Jürgens U (2009). The neural control of vocalization in mammals: a review.. J Voice.

[b67] Jürgens U, von Cramon DYC (1982). On the role of the anterior cingulate cortex in phonation – a case report.. Brain Lang.

[b68] Kaltwasser MT (1990). Acoustic signaling in the black rat (*Rattus rattus*.. J Comp Psychol.

[b69] Kaminski J, Call J, Fischer J (2004). Word learning in a domestic dog: evidence for ‘fast mapping’.. Science.

[b70] Knutson B, Burgdorf J, Panksepp J (2002). Ultrasonic vocalizations as indices of affective states in rats.. Psychol Bull.

[b71] Konopka G, Bomar JM, Winden K, Coppola G, Jonsson ZO, Gao FY, Peng S, Preuss TM, Wohlschlegel JA, Geschwind DH (2009). Human-specific transcriptional regulation of CNS development genes by FOXP2.. Nature.

[b72] Lai CSL, Fisher SE, Hurst JA, Vargha-Khadem F, Monaco AP (2001). A forkhead-domain gene is mutated in a severe speech and language disorder.. Nature.

[b73] Langmore NE (1998). Functions of duet and solo songs of female birds.. Trends Ecol Evol.

[b74] Lee RB, DeVore I (1968). Man the Hunter.

[b75] Li G, Wang J, Rossiter SJ, Jones G, Zhang S (2007). Accelerated FoxP2 evolution in echolocating bats.. PLoS One.

[b76] Lieberman P (2002). On the nature and evolution of the neural bases of human language.. Am J Phys Anthropol Suppl.

[b77] Lieberman P (2006). Toward an Evolutionary Biology of Language.

[b78] Lieberman P, Blumstein SE (1988). Speech Physiology, Speech Perception, and Acoustic Phonetics.

[b79] Lim MM, Young LJ (2006). Neuropeptidergic regulation of affiliative behavior and social bonding in animals.. Horm Behav.

[b80] Maggio JC, Whitney G (1985). Ultrasonic vocalizing by adult female mice (*Mus musculus*.. J Comp Psychol.

[b81] Moles A, Costantini F, Garbugino L, Zanettini C, D’Arnato FR (2007). Ultrasonic vocalizations emitted during dyadic interactions in female mice: a possible index of sociability?. Behav Brain Res.

[b82] Musolf K, Hoffmann F, Penn DJ (2009). Ultrasonic courtship vocalizations in wild house mice, *Mus musculus musculus*. Anim Behav.

[b83] Neumann ID (2008). Brain oxytocin: a key regulator of emotional and social behaviours in both females and males.. J Neuroendocrinol.

[b84] Nietschke W, Bell RW, Zachman T (1972). Distress vocalization of young in three inbred strains of mice.. Dev Psychobiol.

[b85] Nyby JG, Williott JF (2001). Auditory communication among adults. Handbook of Mouse Auditory Research: From Behavior to Molecular Biology.

[b86] Payne RS, McVay S (1971). Songs of humpback whales.. Science.

[b87] Pedersen CA, Vadlamudi SV, Boccia ML, Amico JA (2006). Maternal behavior deficits in nulliparous oxytocin knockout mice.. Genes Brain Behav.

[b88] Pfefferle D, Brauch K, Heistermann M, Hodges JK, Fischer J (2008). Female Barbary macaque (*Macaca sylvanus*copulation calls do not reveal the fertile phase but influence mating outcome.. P Roy Soc Lond B Bio.

[b89] Pika S, Liebal K, Call J, Tomasello M (2005). The gestural communication of apes.. Gesture.

[b90] Pomerantz SM, Nunez AA, Bean NJ (1983). Female behavior is affected by male ultrasonic vocalizations in house mice.. Physiol Behav.

[b91] Radyushkin K, Hammerschmidt K, Boretius S, Varoqueaux F, El-Kordi A, Ronnenberg A, Winter D, Frahm J, Fischer J, Brose N, Ehrenreich H (2009). Neuroligin-3-deficient mice: model of a monogenic heritable form of autism with an olfactory deficit.. Genes Brain Behav.

[b92] Rendall D, Seyfarth RM, Cheney DL, Owren MJ (1999). The meaning and function of grunt variants in baboons.. Anim Behav.

[b93] Roberts LH (1975). The rodent ultrasound production mechanism.. Ultrasonics.

[b94] Russ JM, Jones G, Mackie IJ, Racey PA (2004). Interspecific responses to distress calls in bats (Chiroptera: Verspertilionidae): a function for convergence in call design?. Anim Behav.

[b95] Sales GD (1972). Ultrasound and aggressive behaviour in rats and other small mammals.. Anim Behav.

[b96] Sales GD, Smith JC (1978). Comparative studies of the ultrasonic calls of infant murid rodents.. Dev Psychobiol.

[b97] Scattoni ML, Gandhy SU, Ricceri L, Crawley JN (2008a). Unusual repertoire of vocalizations in the BTBR T+tf/J mouse model of autism.. PLoS One.

[b98] Scattoni ML, McFarlane HG, Zhodzishsky V, Caldwell HK, Young WS, Ricciri L, Crawley JN (2008b). Reduced ultrasonic vocalizations in vasopressin 1b knockout mice.. Behav Brain Res.

[b99] Scattoni ML, Crawley J, Ricceri L (2009). Ultrasonic vocalizations: a tool for behavioural phenotyping of mouse models of neurodevelopmental disorders.. Neurosci Biobehav R.

[b100] Scheuber H, Jagot A, Brinkhof MW (2004). Female preference for multiple condition-dependent components of a sexually selected signal.. P Roy Soc Lond B Bio.

[b101] Schultz W (2006). Behavioral theories and the neurophysiology of reward.. Annu Rev Psychol.

[b102] Schwarting RKW, Jegan N, Wöhr M (2007). Situational factors, conditions, and individual variables which can determine ultrasonic vocalizations in male adult wistar rats.. Behav Brain Res.

[b103] Searcy WA, Marler P (1981). A test for responsiveness to song structure and programming in female sparrows.. Science.

[b104] Sewell GD (1967). Ultrasound in adult rodents.. Nature.

[b105] Sewell GD (1970). Ultrasonic communication in rodents.. Nature.

[b106] Seyfarth RM, Cheney DL, Snowdon CT, Hausberger M (1997). Some features of vocal development in nonhuman primates. Social Influences on Vocal Development.

[b107] Seyfarth RM, Cheney DL (2003). Meaning and emotion in animal vocalizations.. Ann Ny Acad Sci.

[b108] Seyfarth RM, Cheney DL, Marler P (1980). Monkey responses to three different alarm calls: evidence of predator classification and semantic communication.. Science.

[b109] Shepard KN, Liu RC Experience restores innate female preference for male calls. Genes Brain Behav, Oxford.

[b110] Shu WG, Cho JY, Jiang YH, Zhang MH, Weisz D, Elder GA, Schmeidler J, De Gasperi R, Sosa MAG, Rabidou D, Santucci AC, Perl D, Morrisey E, Buxbaum JD (2005). Altered ultrasonic vocalization in mice with a disruption in the Foxp2 gene.. Proc Natl Acad Sci USA.

[b111] Sutton D, Larson CR, Lindeman RC (1974). Neocortical and limbic lesion effects on primate phonation.. Brain Res.

[b112] Számadó S, Hurford JR, Bishop D, Deacon T, d'Errico F, Fischer J, Okanaya K, Szathmáry E, White S, Bickerton D, Szathmáry E (2009). What are the possible biological and genetic foundations for syntactic phenomena?. Biological Foundations and Origin of Syntax.

[b113] Teichmann M, Gaura V, Demonet JF, Supiot F, Delliaux M, Verny C, Renou P, Remy P, Bachoud-Levi AC (2008). Language processing within the striatum: evidence from a PET correlation study in Huntington's disease.. Brain.

[b114] Teramitsu I, White S (2006). FoxP2 regulation during undirected singing in adult songbirds.. J Neurosci.

[b115] Teramitsu I, Kudo LC, London SE, Geschwind DH, White SA (2004). Parallel FoxP1 and FoxP2 expression in songbird and human brain predicts functional interaction.. J Neurosci.

[b116] Teramitsu I, Poopatanapong A, Torrisi S, White SA (2010). Striatal FoxP2 is actively regulated during songbird sensorimotor learning.. PLoS One.

[b117] Tettamanti M, Moro A, Messa C, Moresco RM, Rizzo G, Carpinelli A, Matarrese M, Fazio F, Perani D (2005). Basal ganglia and language: phonology modulates dopaminergic release.. Neuroreport.

[b118] The Autism Genome Project Consortium (2007). Mapping autism risk loci using genetic linkage and chromosomal rearrangements.. Nat Genet.

[b119] Tomasello M (2003). Constructing a Language–A Usage Based Theory of Language Acquisition.

[b120] Uematsu A, Kikusui T, Kihara T, Harada T, Kato M, Nakano K, Murakami O, Koshida N, Takeuchi Y, Mori Y (2007). Maternal approaches to pup ultrasonic vocalizations produced by a nanocrystalline silicon thermo-acoustic emitter.. Brain Res.

[b121] Ullman MT (2001). A neurocognitive perspective on language: the declarative/procedural model.. Nat Rev Neurosci.

[b122] Wang H, Liang S, Burgdorf J, Wess J, Yeomans J (2008). Ultrasonic vocalizations induced by sex and amphetamine in M2, M4, M5 muscarinic and D2 dopamine receptor knockout mice.. PLoS One.

[b123] Whitney G, Cobte JR, Stockton MD, Tilson EF (1973). Ultrasonic emission: do they facilitate courtship in mice?. J Comp Physiol Psychol.

[b124] Wild M (2008). Tierphilosophie zur Einführung.

[b125] Wilson TR, Hare JF (2004). Animal communication: ground squirrel uses ultrasonic alarms.. Nature.

[b126] Winslow JT, Hearn EF, Ferguson J, Young LJ, Matzuk MM, Insel TR (2000). Infant vocalization, adult aggression, and fear behaviour of an OXT null mutant mouse.. Horm Behav.

[b127] Zhang JZ, Webb DM, Podlaha O (2002). Accelerated protein evolution and origins of human-specific features: FOXP2 as an example.. Genetics.

[b128] Zuberbühler K, Cheney DL, Seyfarth RM (1999). Conceptual semantics in a nonhuman primate.. J Comp Psychol.

